# Employing Deep-Learning Approach for the Early Detection of Mild Cognitive Impairment Transitions through the Analysis of Digital Biomarkers

**DOI:** 10.3390/s23218867

**Published:** 2023-10-31

**Authors:** Rajaram Narasimhan, Muthukumaran Gopalan, Mohamed Yacin Sikkandar, Ahmad Alassaf, Ibrahim AlMohimeed, Khalid Alhussaini, Adham Aleid, Sabarunisha Begum Sheik

**Affiliations:** 1 Centre for Sensors and Process Control, Hindustan Institute of Technology and Science, Chennai 603103, India; gmkumaran@hindustanuniv.ac.in; 2 Department of Medical Equipment Technology, College of Applied Medical Sciences, Majmaah University, Al Majmaah 11952, Saudi Arabia; am.alassaf@mu.edu.sa (A.A.); i.almohimeed@mu.edu.sa (I.A.); 3 Department of Biomedical Technology, College of Applied Medical Sciences, King Saud University, Riyadh 12372, Saudi Arabia; kalhussaini@ksu.edu.sa (K.A.); adaleid@ksu.edu.sa (A.A.); 4 Department of Biotechnology, P.S.R. Engineering College, Sivakasi 626140, India; sabarunishabegum@psr.edu.in

**Keywords:** AD progression, MCI transition, daily activities, deep learning, RNN LSTM, time series statistic, digital biomarkers

## Abstract

Mild cognitive impairment (MCI) is the precursor to the advanced stage of Alzheimer’s disease (AD), and it is important to detect the transition to the MCI condition as early as possible. Trends in daily routines/activities provide a measurement of cognitive/functional status, particularly in older adults. In this study, activity data from longitudinal monitoring through in-home ambient sensors are leveraged in predicting the transition to the MCI stage at a future time point. The activity dataset from the Oregon Center for Aging and Technology (ORCATECH) includes measures representing various domains such as walk, sleep, etc. Each sensor-captured activity measure is constructed as a time series, and a variety of summary statistics is computed. The similarity between one individual’s activity time series and that of the remaining individuals is also computed as distance measures. The long short-term memory (LSTM) recurrent neural network is trained with time series statistics and distance measures for the prediction modeling, and performance is evaluated by classification accuracy. The model outcomes are explained using the SHapley Additive exPlanations (SHAP) framework. LSTM model trained using the time series statistics and distance measures outperforms other modeling scenarios, including baseline classifiers, with an overall prediction accuracy of 83.84%. SHAP values reveal that sleep-related features contribute the most to the prediction of the cognitive stage at the future time point, and this aligns with the findings in the literature. Findings from this study not only demonstrate that a practical, less expensive, longitudinal monitoring of older adults’ activity routines can benefit immensely in modeling AD progression but also unveil the most contributing features that are medically applicable and meaningful.

## 1. Introduction

Alzheimer’s disease (AD) is a neurodegenerative disease that predominantly affects older adults aged 65 and above. The progression of this degeneration can span as long as 30 years. Until now, this condition has been irreversible, and the cause and cure for this condition are under intense research by the medical community [1]. Mild cognitive impairment (MCI) is known to be a transitional stage between the preclinical stage and advanced AD stage. In the preclinical stage, certain physiological changes start evolving, but no noticeable symptom is observed in older adults. In the MCI stage, older adults start presenting symptoms of cognitive decline in terms of memory, executive function, attention, etc., but no functional impairment exists. It is also to be noted that the MCI condition does not always lead to the AD stage [2]. Though older adults in the MCI stage are not dependent on others to perform day-to-day tasks, the rate of cognitive decline varies from one individual to another. In the advanced stage of AD, symptoms of cognitive, behavioral, and functional impairment are evident, and older adults are completely dependent on others even for basic needs [3]. Therefore, it is crucial to detect the transitional point to the MCI stage (i.e., the pre-symptomatic stage of AD) as early as possible and provide appropriate interventions to delay the onset or progression [1]. As the progression of this neurodegeneration occurs over time, the impact of this degeneration manifests in changes in the daily routines of older adults. A growing body of the literature also indicates that behavioral, functional, and cognitive changes continue to occur before any symptoms of AD are detected clinically [4,5,6]. Several other studies prove that the daily activities of living (ADLs) or daily routines, if observed effectively, can provide appropriate measures to detect the above-said changes and, thus, detect the transition to the MCI stage [3,7,8,9,10]. Advancements in smart sensing technology enable continuous monitoring of the daily routines of older adults in a naturalistic environment (e.g., their residences) and offer a new class of indicators, called digital biomarkers [11,12], to detect the transition to the MCI stage. Digital biomarkers, in this study’s context, represent the objective, quantifiable behavioral/functional data derived from environmental/ambient sensors via continuous monitoring of daily routines, and a few examples include walking speed, sleep duration, etc. It is understood that one’s performance at any single point in time may not reveal the best indicators of cognitive decline but rather by observing the activity trend over a long period of time [13]. Continuous assessment of these digital biomarkers enables not only the observation of subtle changes in routines but also the understanding of intra-individual variability in activity patterns, which are key to detecting the transition to the MCI stage. Due to the degenerative nature of AD, the disease state at a particular time point is not independent of the state at previous time points. This informs the significance of a better approach to analyze the continuously gathered patient data rather than analysis of data at a single point in time [13]. This approach can be corroborated by the continuous monitoring of the older adults’ activity routines via sensors, resulting in longitudinal data, which comprises various activity measures (afore-mentioned digital biomarkers). Construing this longitudinal data as time series data and analyzing them further becomes a natural choice for AD progression modeling. Time series data, exhibiting the temporal pattern, are often complex in nature, contrary to static data, and pose challenges like being noisy, irregular, etc. Extracting valuable information from the temporal data using innovative approaches that transcend the boundaries of several domains (e.g., statistics, time series analysis, signal processing) has been the topic of active research [14,15]. The activity time series data, in its original form, is a numeric series of particular activity measure values (e.g., walking speed). Abstracting the time-series statistic from a numeric time series of a particular activity will be a useful characterization of that activity and can provide better indicators of pattern/trend changes for the progression modeling.

Since this degeneration progresses over time, an inherent temporal/sequential nature can be seen in this progression. Hence, any technique to model this disease progression should take this sequential nature into account and leverage this effectively for making predictions. Specifically, this is important in the scenario of predicting the progression at a future time point, given the continuously acquired activity trend data points. Being a special type of neural network, the recurrent neural network (RNN) can pass temporal information from one time step to the next time step, due to which it can recognize the patterns in sequential input data and predict a sequence of outputs. An extension of the plain RNN, Long short-term memory (LSTM) RNN consists of LSTM cells, which are building units of the network, and these cells enable RNNs to capture long-term dependencies that exist in sequential input. Therefore, the LSTM RNN is an apt choice to model the AD progression by leveraging the temporal patterns found in activity trend data [16]. The activity trend data characterized by time series statistics as input to the LSTM network will further reinforce their prediction skill along with their natural ability to recognize the long-term dependencies in sequential data.

Detection or prediction of AD condition leveraging various modalities (e.g., neuroimaging, cognitive assessment scores, genetic, neuropathology) as well as computational methods (e.g., regression, decision trees, Bayesian networks, neural networks) has been a growing field of research. As discussed earlier, neurodegeneration associated with AD lets this progression be inherently temporal/sequential in nature. Hence, it is evident that this temporal/sequential nature, if leveraged properly, will result in an effective AD progression modeling. Most appropriate to the proposed work in this study is to survey the other works that have considered this temporal/sequential nature for AD progression modeling. Wang et al. [17] proposed a deep-learning approach (RNN LSTM) to predict AD progression by utilizing individuals’ multiple visits data that were captured longitudinally. These visit data, supplied by the National Alzheimer’s Coordinating Center (NACC), consisted of demographics, health history, physical parameters, and neuropsychiatric assessment scores. In another study, Fouladvand et al. [18] modeled the prediction of MCI transition through RNN LSTM using the Electronic Health Records (EHR) data obtained from the Mayo Clinic Study on Aging (MCSA). Clinical notes in unstructured format were the predominant source in deriving the features related to disorders, Neuropsychiatric symptoms, and cognitive functioning. Their study was able to demonstrate a good potential for incorporating temporal EHR patterns and predicting the MCI transition. In modeling the AD progression, El-Sappagh et al. [19] developed a deep-learning approach, which was a combination of the Convolutional Neural Network (CNN) and LSTM network. They utilized data from multiple modalities such as neuroimaging, cognitive scores, neuropathology, and neuropsychiatric assessment scores obtained from the Alzheimer’s Disease Neuroimaging Initiative (ADNI) database. In their approach, they construed each modality data as a time series, derived temporal features from each time series, and fused those multi-modal features to train their deep-learning model.

Abuhmed et al. [20] developed and examined a hybrid deep-learning model for AD progression detection, in which the deep features exhibiting temporal connections were extracted by the LSTM model and subsequently used to train several traditional classifiers. Their approach fused the multi-modal time series data obtained from the ADNI database that included neuroimaging, cognitive scores, neuropathology, and neuropsychiatric assessment data. In their study, Mukherji et al. [21] aimed to identify non-invasive, inexpensive markers to predict AD progression and investigated the utility of neuropsychological test scores alone in training the LSTM-based model. These test scores, obtained from ADNI, were captured at multiple visits per patient and formed a longitudinal data input for LSTM to infer the temporal connections. Pang et al. [22] investigated if a small set of clinical variables could help predict the transition from normal cognition to MCI by leveraging the NACC dataset. Data used in their study included individual baseline and follow-up visits data, forming a longitudinal view that consisted of demographics, health history, and neuropsychiatric scores. All the above-mentioned studies were able to leverage the temporal nature of the AD progression by way of representing data in a longitudinal format, and that longitudinal data would essentially be a collection of features/measures gathered at specific time points (e.g., initial or follow up visit to the clinic, in-patient hospital stay). These data did not represent continuously acquired data but rather point-in-time evaluations/assessments.

Furthermore, most of these studies utilized expensive, invasive modalities, such as neuroimaging and neuropathology, to identify appropriate markers in progression modeling. Moreover, some of these point-in-time data (cognitive/functional assessment scores) would depend on self-reported or informant-reported responses, which would most likely pose challenges like not being completely objective or inter-assessor variability. In consideration of these challenges, there has been a continuous need for an alternate approach that can be inexpensive, non-invasive, and more objective in nature to acquire relevant markers that can effectively predict AD progression. As discussed in the previous section, digital biomarkers derived from older adults’ daily routines via continuous monitoring offer the best alternate approach, and these can be effective in observing intra-individual activity pattern changes since these are gathered as the older adults carry out their routines in a naturalistic environment (e.g., residences).

In exploring the adoption of the above-mentioned approach for AD progression modeling, this study seeks to find answers to the following questions:

- Can the activity measures computed from longitudinal monitoring of older adults’ daily routines via ambient sensors predict AD progression, specifically the transition to the MCI stage, at a future time point?
- Can the activity trends characterized by time series statistics provide fine-grain details of an individual’s activity trends and aid the prediction model to perform better?
- Which activity features/measures contribute most to the prediction of the MCI transition at a future time point?

We hypothesize that the activity trend data captured from daily routines and characterized by time series summary statistic features provide sufficient information to predict the MCI transition at a future time point.

## 2. Materials and Methods

**Figure 1:**
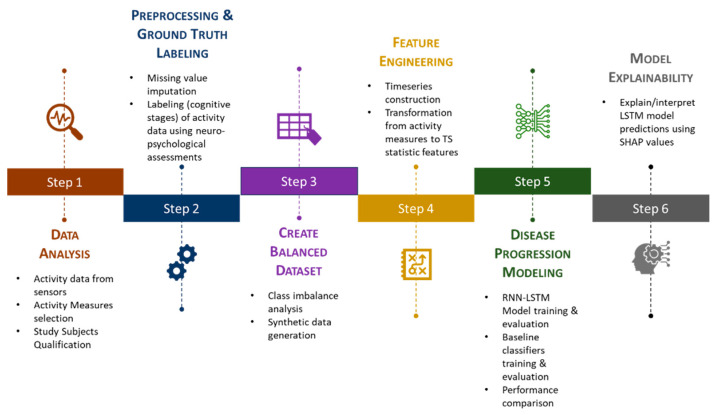
Workflow of the proposed study.

In this study, the MCI transition prediction model was developed based on the state-of-the-art deep-learning technique RNN LSTM. The prediction performance of the proposed deep-learning model was compared against the traditional machine-learning classifiers such as Support Vector Machines (SVM) and Random Forest (RF) models. To evaluate the performance of the proposed model, this study adopted a commonly used metric, “overall prediction accuracy”, as well as three more custom-defined metrics, namely, “degenerating identification accuracy”, “transition identification accuracy”, and “MCI identification accuracy”. To ensure that the model is not only accurate but also its outcomes are explainable and clinically meaningful, this study adopted the SHapley Additive exPlanations (SHAP) framework. Figure 1 depicts the end-to-end workflow of the approach followed in this study. It comprises six high-level steps, starting from the data acquisition step through the model explanation step.

Step 1—Data analysis is performed to understand the activity data captured through ambient sensors and qualify appropriate subjects and activity measures based on availability;

Step 2—Preprocessing of data is carried out in terms of imputing missing values. By leveraging various neuropsychological test scores captured at periodic assessment points, each subject’s activity data are assigned with ground truth labels (i.e., cognitive stage);

Step 3—Analysis of imbalance in a dataset in terms of cognitive stage labels/classes. Simulating several subjects’ activity data in their entirety using special techniques to address imbalance issues;

Step 4—Each activity measure is represented as a time series and several time series statistic features are derived to represent the activity trend that would be the input to the model;

Step 5—The RNN LSTM model is built, trained, and tested for its prediction performance as well as compared against baseline classifiers;

Step 6—Using the SHAP framework, model outcomes are explained and interpreted.

### 2.1. Data Acquisition and Analysis

**Figure 2:**
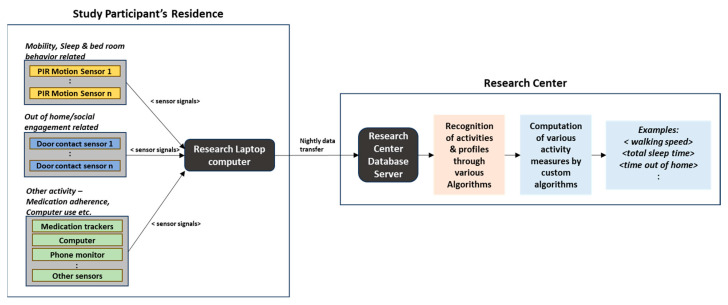
Data acquisition through sensors.

The dataset used in this study is provided by the Oregon Center for Aging and Technology (ORCATECH) at the Oregon Health and Science University (OHSU) [13]. As part of a longitudinal community cohort study, ORCATECH deployed an unobtrusive home-based activity assessment platform at hundreds of seniors’ homes in the existing community, and this platform included several ambient sensors to monitor and capture daily activity patterns on a continuous basis. Passive infrared (PIR) motion sensors and wireless contact switches were those ambient sensors, to name a few. Activity data captured typically corresponded to ADLs (Activities of Daily Living)/iADLs (instrumental Activities of Daily Living) and included activities pertaining to physical mobility, sleep, room transitions, out-of-home visits, etc. Figure 2 depicts the block schematic of a typical data acquisition process from a study participant’s residence and further data processing by the research center [23].

In addition to continuous sensor-based monitoring, annual clinical assessments of study subjects were performed by qualified health professionals. Annual clinical assessment data typically included demographics, functional assessment scores, health assessments, and scores from a battery of neuropsychological tests such as Trail Making test, Category Fluency, Memory recall, and so on. Inclusion criteria for the study subjects were the age of 70 years and above (at the time of onboarding), cognitively healthy (measured by Clinical Dementia Rating—CDR < 0.5 and Mini-Mental State Examination—MSME score > 24), and with an age-appropriate health condition. For further details on participants’ recruitment criteria, clinical assessments, in-home sensor modalities, and activity measures calculations, readers can refer to [13,23]. Thus, the dataset used in this study includes both sensor-captured activity measures and clinical assessment data. Activity measures correspond to the monitoring period from 2015 to 2018. Activity data supplied by ORCATECH contained the day-level activity measures (per subject) derived from raw sensor signals. ORCATECH applied various proprietary activity recognition algorithms on raw sensor signal data and derived these measures (e.g., walking speed, number of walks, sleep duration). There were 125,119 records of day-level sensor data pertaining to the monitoring period ≥ 2015. Based on the analysis, sensor-captured activity records falling under the below scenarios were removed as a clean-up: records that were completely duplicated; records belonging to subjects who did not have corresponding clinical assessment data; records belonging to subjects who were missing activity measures (0 or blank) for the majority of their monitoring duration/timeline (e.g., on average up to 80% of activity records per subject), and records containing “blanks” in almost all activity measures.

After the clean-up, as mentioned above, it resulted in 57,785 records (day-level activity measures) that had to be taken for further processing. This dataset corresponds to a total of 65 unique subjects (older adults).

### 2.2. Data Preprocessing and Ground Truth Labeling

In the qualified activity dataset, as explained in the previous section, there were still missing values for certain activity measures for a few subjects. As mentioned earlier, each activity record was at day-level granularity, and a generic approach would be to fill a missing value with a value found in the previous day’s record or the next day’s record of that subject. This study did not adopt that approach, instead creating a reference dataset consisting of average values per activity measured per subject by week, by month, by quarter, and by year. When a few contiguous days in a week were missing an activity measure, the average value computed for that week of the same subject was used to fill in those missing values; similarly, when a few contiguous weeks in a month were missing an activity measure, average value computed for that month of the same subject was used to fill in those missing values. In this way, the trend of activity measures over time was preserved as much as possible.

**Figure 3:**
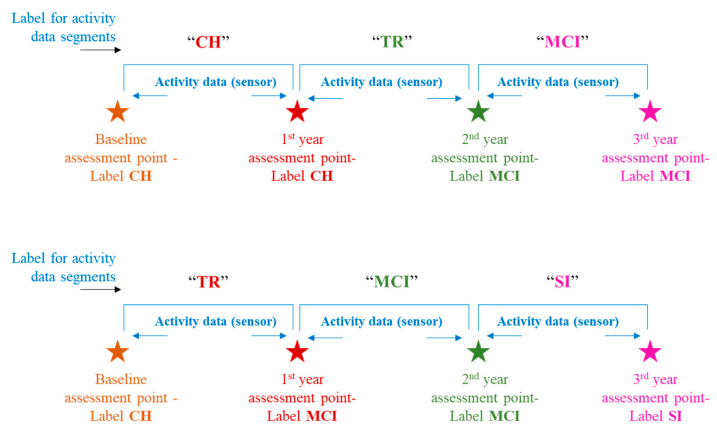
Ground truth labeling with cognitive stage.

This study leveraged clinical assessment data, particularly the neuropsychological test scores, to assign the labels to activity data. These labels indicate a “cognitive stage” of the subject at a particular time in that subject’s activity monitoring duration or activity timeline. Thus, for every subject, there could be more than one cognitive stage label assigned depending on whether that subject remained cognitively intact throughout the monitoring duration or transitioned to the next stage of impairment. Petersen criteria [24], [25] were adopted for cognitive stage labeling, especially the MCI stage. Cognitive stage labels assigned were “Cognitively Healthy” (CH), “Mild Cognitive Impairment” (MCI), and “Severely Impaired” (SI). The transition process of an individual shifting from cognitively healthy to the MCI stage is known to be gradual in nature rather than to occur suddenly. To account for this fact, this study introduces a label called “Transitioning” (TR), and this label would be assigned to the activity data falling in between the CH and MCI stages. “Transitioning” (TR), in this study context, indicates the phase during which an individual begins to undergo a gradual shift from his/her cognitively intact state toward exhibiting signs of decline in one or more domains such as memory, attention, etc. However, the individual continues to maintain his/her functional independence. Depiction of a few labeling scenarios can be seen in Figure 3. For more details on the labeling criteria, readers can refer to [26].

### 2.3. Create a Balanced Dataset

After labeling the activity data with cognitive stages, an analysis was performed to ascertain the distribution of subjects by their cognitive stage transitions. A variety of cognitive stage transitions existed, as follows:

- Forty-seven subjects remained “CH”;
- Two subjects had a transition trajectory of CH- > TR- > MCI;
- Four subjects had a transition trajectory of CH- > TR;
- Two subjects remained at stage TR;
- Two subjects had a transition trajectory of TR- > MCI;
- Eight subjects remained at MCI.

It is quite evident from the above distribution that there existed an imbalance between cognitively healthy subjects vs. cognitively declined/degenerated subjects. To address this imbalance issue, this study adopted the synthetic data generation approach, wherein the activity data corresponding to degenerating cases (i.e., minority cases, as seen above) were used as input to generate a synthetic activity dataset pertaining to new subjects. This study did not opt for over-sampling of minority cases because it would simply result in mere replication of the day-level activity records. Replicating day-level records independently would not be helpful for this study context; rather, this study requires an approach of emulating the entire sequence of activities at a subject level to preserve the temporal nature of the activity trends occurring over time. Hence, this study utilized a Python-based data synthesizer package called “Synthetic Data Vault” (SDV) [27], which uses machine-learning/deep-learning models to learn sequential patterns from real data and emulates them when creating synthetic data. After creating the synthetic activity dataset, a balanced dataset in terms of cognitively healthy vs. degenerated subjects has resulted. Descriptive statistics about the subjects in the complete dataset are presented in Table 1. Based on the cognitive stage transitions during the monitoring period, the study participants are categorized into three groups, namely, “cognitively healthy”, “transitioning to MCI”, and “mild cognitive impairment”. The “cognitively healthy” group includes the participants who remained cognitively normal throughout the monitoring/study period. “Transitioning to MCI” and “mild cognitive impairment” groups include the cognitively degenerated cases. The group “transitioning to MCI” includes the participants who were cognitively normal at the baseline and found to be in the transitioning phase to MCI but did not end up in the MCI stage at the study completion. The third group, “mild cognitive impairment”, includes the participants who were cognitively normal at the baseline and found to be in the MCI stage at the study completion.

As seen in Table 1, the cognitively healthy group consists of 47 participants (transition scenario is CH), the transitioning group consists of 102 participants (transition scenarios are CH- > TR and TR), and the mild cognitive impairment group consists of 148 participants (transition scenarios are CH- > TR- > MCI, TR- > MCI and MCI).

After cleaning up the original data supplied (as explained in Section 2.1), the resulting dataset consisted mostly of female subjects, and that pattern was reflected when synthetic data were created by emulating the cleaned-up dataset. This is, in fact, evident from the gender distribution depicted in Table 1. Subjects of higher age appeared to be in either transition or MCI scenario compared to the cognitively healthy scenario, and it can be seen in both male and female subjects. Also, for these subjects, the average sensor monitoring duration is shorter than for the subjects in the cognitively healthy scenario, and this could mean that the oldest subjects decline relatively faster. Another inference is that subjects with higher education levels exist in both cognitively healthy and degenerating scenarios, while subjects with relatively lower education levels appear in degenerating scenarios.

### 2.4. Data Segmentation and Feature Engineering

Table 2 lists the activity measures that exist in the activity dataset, apart from the demographic details [10]. As stated earlier, these activity measures were derived from the raw signals received from the ambient sensors via continuous monitoring and obtained at day-level granularity per every subject. Cognitive/behavioral domains to which each measure belongs are also mentioned. For every subject, each activity measure captured over the monitoring duration forms an activity sequence.

A key goal of this study is to construe the activity sequence data as a time series and characterize the activity trends by computing appropriate time series (TS) statistics. Toward this goal, a “sliding window” method was adopted to segment activity sequences into multiple windows, and from each window, a set of TS statistics was computed. This process of feature transformation, in other words, the temporal abstraction of time series, enables to retain the temporal trend of the activity [15]. In choosing the size of this sliding window, there were no specific standards found as relevant to this study context. Kaye et al. [13], in their study, used a 4-week snapshot to derive the activity metrics for their analysis. In this study’s context, it was decided to consider neither a very short window nor a wide window. The wide window might capture coarse grain variations in the activity pattern, while the very short window might not be effective in capturing the activity trends. Hence, there needs to be a balance between these two so that the window size should provide an opportunity to capture fine grain variations in activity patterns as well as accommodate cycles of routine activities (e.g., daily/weekly cycles). Thus, it was decided to use a window size of 10 days (a week and a half) with an overlap of 75% between consecutive windows (that means a skip size of approximately 3 days). A Python-based package called “TSFRESH” was used to extract the TS statistic measures. TSFRESH package offers systematic time-series feature extraction by various algorithms from domains like statistics, time-series analysis, signal processing, etc. [28].

Table 3 lists various TS statistic measures leveraged in this study, along with the significance of each of those measures. It is to be noted that these 6 TS statistics were extracted from each of the original activity feature sequences.

**Figure 4:**
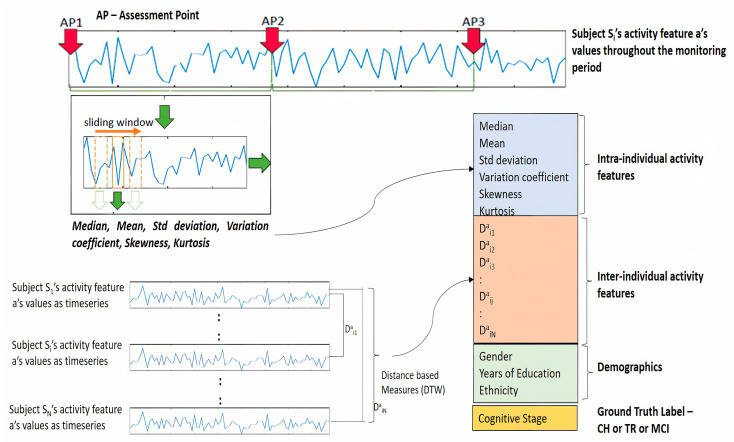
Illustration of feature extraction for a specific subject and activity sequence.

TS statistic features computed for each subject’s activity data indicate the intra-individual activity trends. To provide more context to the prediction model, it is also necessary to understand how an individual’s activity trend looks like to other individuals in the study cohort considered. This relative positioning can be derived by computing distance-based measures between individuals’ activity time series. These measures indicate inter-individual activity trends. “Dynamic Time Warping” (DTW) is one such method that helps compute the distance measure between two time series and provides more context in terms of similarity/dissimilarity between time series. Also, DTW distance measures, when used as features in machine learning, have been proven to strengthen the TS classification tasks [29]. DTW distance measures were computed between the original activity sequence/time series. Thus, for every subject, DTW distances between each activity time series and that of the remaining subjects become features for the prediction model, in addition to TS statistic features and demographics. The computation of DTW distance measures in this study was performed by using a Python package “dtw-Python” [30,31]. Figure 4 depicts the overall feature engineering process explained in previous sections. This figure illustrates the extraction of TS statistics from the sequence of the activity “a” belonging to subject Si as well as DTW distance measures between a’s sequence of Si and the rest of the subjects like S1, S2, … SN.

The mathematical formulation of the above-mentioned feature extraction and the construction of input data to the model is described below [14,15]. Let us consider subject Si, who is part of the cohort of N subjects considered for this study, and this cohort is represented as

S = {S1, S2, …, Si, …, SN}(1)

The activity dataset of subject Si consists of day-level activity measures and is represented as a matrix

(2) $$S_{i}^{AM}=\left[\begin{array}{ccccc} d_{1} & a_{i1} & b_{i1} & \ldots & m_{i1}\\ d_{2} & a_{i2} & b_{i2} & \ldots & m_{i2}\\ \ldots & \ldots & \ldots & \ldots & \ldots \\ d_{t} & a_{it} & b_{it} & \ldots & m_{it} \end{array}\right]$$

where “d” indicates the day of monitoring, and this dataset consists of “t” number of days of observation, and <a, b, …, m> indicate the activity measures. Thus, this subject Si has “m” number of activity measures captured for “t” number of days.

As discussed earlier, each activity measure forms a sequence and can be construed as a time series, such as

(3) $$a_{i}=\left\{a_{i1},a_{i2},\ldots ,a_{it}\right\}$$

(4) $$b_{i}=\left\{b_{i1},b_{i2},\ldots ,b_{it}\right\}$$

and so on. The next step is to extract the TS statistic features from each of these activity time series by following the sliding window method. Assuming “k” number of windows are generated and, in each window, the six TS statistic features (Table 3) are extracted per each activity time series, and the resulting feature matrix for subject Si will look like

(5) $$S_{i}^{FM}=\left[\begin{array}{ccccccccc} w_{1} & f_{i1}^{a1} & f_{i1}^{a2} & f_{i1}^{a3} & f_{i1}^{a4} & f_{i1}^{a5} & f_{i1}^{a6} & \ldots & f_{i1}^{m6}\\ w_{2} & f_{i2}^{a1} & f_{i2}^{a2} & f_{i2}^{a3} & f_{i2}^{a4} & f_{i2}^{a5} & f_{i2}^{a6} & \ldots & f_{i2}^{m6}\\ \ldots & \ldots & \ldots & \ldots & \ldots & \ldots & \ldots & \ldots & \ldots \\ w_{k} & f_{ik}^{a1} & f_{ik}^{a2} & f_{ik}^{a3} & f_{ik}^{a4} & f_{ik}^{a5} & f_{ik}^{a6} & \ldots & f_{ik}^{m6} \end{array}\right]$$

where $f_{ik}^{a1}$ refers to the first TS statistic feature extracted from the window $w_{k}$ of the activity sequence “a” of the subject Si; similarly, $f_{ik}^{a6}$ refers to the 6th TS statistic feature extracted from the window $w_{k}$ of the activity sequence “a” of the subject Si.

After extracting the TS statistic features, DTW distance measures are calculated for the original activity sequence/time series. Let us consider the “N” number of sequences for activity “a” where N is the number of subjects in this study, and these sequences can be expressed as

(6) $$a_{1}=\left\{a_{11},a_{12},\ldots ,a_{1t}\right\}\;a_{2}=\left\{a_{21},a_{22},\ldots ,a_{2t}\right\}\;\ldots \ldots \ldots \ldots \ldots \;a_{N}=\left\{a_{N1},a_{N2},\ldots ,a_{Nt}\right\}$$

Please note that the sequences shown above appear to be equal in length (“t” time points); however, it is not necessary for all subjects to have equal length of activity sequence, and the very purpose of the DTW method is to find the distance between varying length of time series. For the given subject Si, the distance measures with respect to activity sequence “a” will be as follows:

$D_{i1}^{a}\to \mathrm{distance}\;\mathrm{between}\;\mathrm{activity}\;\mathrm{sequences}\;\mathrm{of}\;S_{1}\;\mathrm{and}\;S_{i};$

$D_{i2}^{a}\to \mathrm{distance}\;\mathrm{between}\;\mathrm{activity}\;\mathrm{sequences}\;\mathrm{of}\;S_{2}\;\mathrm{and}\;S_{i}\;\mathrm{and}\;\mathrm{so}\;\mathrm{on}.$

The distance measures for all remaining activity sequences are also computed and the resulting distance measures for subject Si will be as follows:

(7) $$\left[\langle D_{i1}^{a},D_{i2}^{a},\ldots ,D_{iN}^{a}\rangle \langle D_{i1}^{b},D_{i2}^{b},\ldots ,D_{iN}^{b}\rangle \ldots .\langle D_{i1}^{m},D_{i2}^{m},\ldots ,D_{iN}^{m}\rangle \right]$$

The measures in (7) are appended in all rows in the dataset (5) along with the demographic data (age, gender, and years of education), and the resulting dataset belonging to subject Si will be the input data to the model. The resulting dataset can be expressed in simplified form for an easy understanding as follows:

(8) $$S_{i}^{FM}=\left[\begin{array}{cccc} w_{1} & \langle f_{i1}^{a1},f_{i1}^{a2},\ldots ,f_{i1}^{m5},f_{i1}^{m6}\rangle & \langle D_{i1}^{a},{\ldots ,D}_{iN}^{a},\ldots ,D_{iN}^{m}\rangle & \langle {age}_{i},{gen}_{i},{edu}_{i}\rangle \\ w_{2} & \langle f_{i2}^{a1},f_{i2}^{a2},\ldots ,f_{i2}^{m5},f_{i2}^{m6}\rangle & \langle D_{i1}^{a},{\ldots ,D}_{iN}^{a},\ldots ,D_{iN}^{m}\rangle & \langle {age}_{i},{gen}_{i},{edu}_{i}\rangle \\ \ldots & \ldots & \ldots & \ldots \\ w_{k} & \langle f_{ik}^{a1},f_{ik}^{a2},\ldots ,f_{ik}^{m5},f_{ik}^{m6}\rangle & \langle D_{i1}^{a},{\ldots ,D}_{iN}^{a},\ldots ,D_{iN}^{m}\rangle & \langle {age}_{i},{gen}_{i},{edu}_{i}\rangle \end{array}\right]$$

As seen in (8), each row is a time step comprising TS statistic features, distance features, and demographics, and it conforms to the time step requirement of the LSTM model. Feature variables in (8) can further be represented in a simplified form for readability, as follows:

(9) $$S_{i}^{FM}=\left[\begin{array}{cc} w_{1} & \langle X_{1}^{i}\rangle \\ w_{2} & \langle X_{2}^{i}\rangle \\ \ldots & \ldots \\ w_{k} & \langle X_{k}^{i}\rangle \end{array}\right]$$

where $X_{1}^{i}$ represents the feature vector as at the time step $w_{1}$ for the subject Si.

Based on the ground truth labeling, as explained in the previous section, the label vector is formed, and that would consist of a label/class corresponding to each time window. The label vector for subject Si is expressed as follows:

(10) $$Y_{i}=\left[y_{1}^{i},y_{2}^{i},y_{3}^{i},\ldots ,y_{k}^{i}\right]$$

where $y_{j}^{i}\in \{CH,TR,MCI\}$.

The labels are encoded as one hot vector later in the LSTM model training phase. The goal of this study is to predict the cognitive stage/label at a future time point, given the activity features captured at historical time points. Time step “$w_{k}$” is considered as the future time point at which the label “$y_{k}^{i}$” needs to be predicted using the activity features $\langle X_{1}^{i}\rangle ,\langle X_{2}^{i}\rangle ,\ldots ,\langle X_{k-1}^{i}\rangle$ captured at time points $w_{1},w_{2},\ldots .,w_{k-1}$, respectively.

Thus, for subject $S_{i}$, the label to predicted can be expressed as

(11) $${\tilde{y}}_{k}^{i}=f(\langle X_{1}^{i}\rangle ,\langle X_{2}^{i}\rangle ,\ldots ,\langle X_{k-1}^{i}\rangle )$$

Above computations are repeated for all other subjects to arrive at feature matrices such as {$S_{1}^{FM}$, $S_{2}^{FM}$, $S_{3}^{FM}$, …., $S_{N}^{FM}$} and label vectors such as, {$Y_{1},Y_{2},Y_{3},\ldots .,Y_{N}\}$.

### 2.5. LSTM-Based Disease Progression Modeling

The prediction by the proposed LSTM model is set to be a classification task with multi-class labels as targets (CH, TR, and MCI). As seen in (8), the feature matrix for a given subject consists of time-sequenced features, and when these time-sequenced data are given as input to the LSTM, it recognizes the temporal connections or dependencies in the input sequence (historical time points) and learns to predict the cognitive stage/label at a future time point.

**Figure 5:**
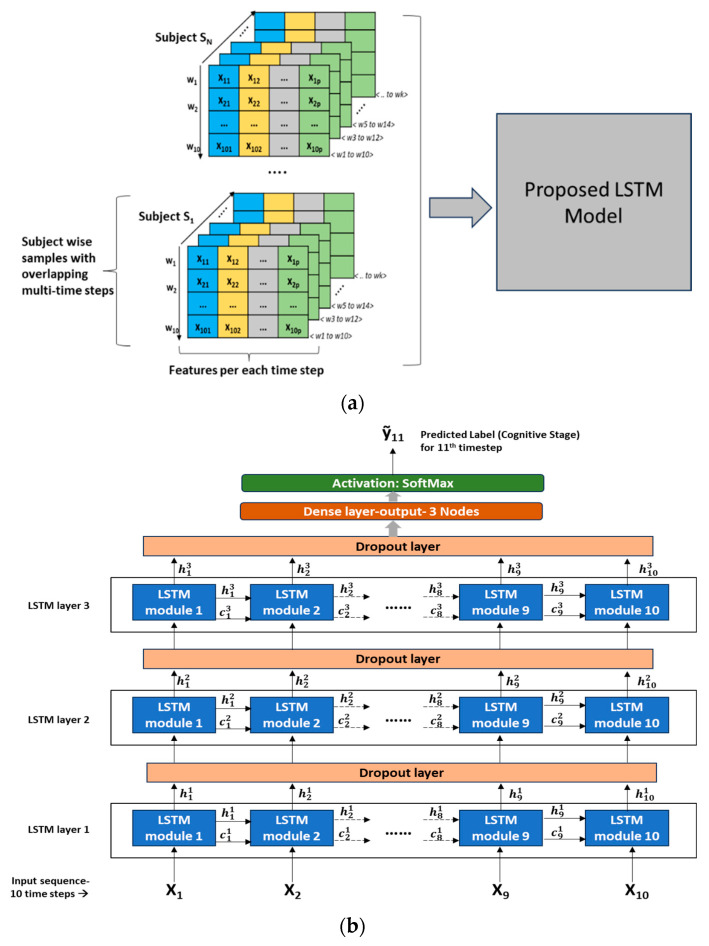
(**a**) Illustration of subject-wise, multi-time stepped samples for training. (**b**) LSTM neural network architecture—input layer with 10 timesteps and output layer with 3 nodes.

Model training approach: In the overall dataset across all subjects, a wide variety of cognitive transition trajectories were seen, such as CH, CH- > TR, CH- > TR- > MCI, TR- > MCI, TR, and MCI. It was decided to expose all these transition paths to the LSTM model so that the model could learn the nuances associated with each of these transition scenarios. Hence, in every subject’s dataset (time-sequenced data), a split of 75%:15%:10% was applied to create a training, validation, and testing dataset, respectively. Tensors of feature datasets were created as input to the LSTM model. Since LSTM input needs to be in the format of <samples, time steps, features>, several samples with overlapping time steps were created from every subject’s feature matrix (8). Figure 5a depicts the multi-time stepped samples of the model.

Figure 5b depicts the detailed LSTM network architecture with 3 LSTM layers stacked and an output layer consisting of 3 nodes with SoftMax activation. A dropout layer is introduced between the LSTM layers to reduce overfitting and improve the generalization of the model. Detailed hyperparameter configurations of the network are presented in Table 4. This LSTM architecture follows a “many-to-one” pattern, which means that the current prediction task involves many timestep inputs with one predicted label as the output corresponding to a future time point. As seen in Figure 5b, LSTM modules/cells are the building units of the network, which enable RNNs to capture long-term dependencies that exist in sequential input. Each LSTM cell is associated with a hidden state/output and a cell state. The hidden state “$h_{t}^{i}$” denotes the hidden state of “t^th^” LSTM cell present in “i^th^” layer, and similarly, “$c_{t}^{i}$” denotes the cell state of “t^th^” LSTM cell present in “i^th^” layer. The first LSTM cell in the first layer uses the initial state of the RNN and the first timestep of the sequence to compute the first output and the updated cell state. On the “i^th^” layer, at time step “t”, the cell uses the current state of the RNN ($c_{t-1}^{i},h_{t-1}^{i}$) and the next time step of the sequence to compute the output and the updated cell state “$c_{t}^{i}$”. As in Figure 5b, in the stacked layers of LSTM, the hidden state “$h_{t}^{i}$” becomes the input to the LSTM cell corresponding to the same timestep on the “(i + 1) ^th^” layer. Thus, the cell state contains information learned from the previous time steps and preserves it in its memory. At each time step, the layer adds information to or removes information from the cell state, and this operation is regulated through special components called gates. For further details on these gates, the mathematical relationship among the gates, cell states, and output, the reader is referred to [16]. On the output layer, the SoftMax function is used to derive the final output “ỹ_11_” (corresponds to the 11th timestep given the 10 timesteps as input sequence). The SoftMax function will map the non-normalized output to a probability distribution over predicted target labels or cognitive stages.

Initial experiments revealed a value of “10” time steps per sample and an overlap of “8” time steps to create multiple samples per subject would be an optimal combination for further training and testing. The LSTM model was trained and tested for various scenarios to ascertain how the model would perform in predicting the future cognitive stage.

- Model with Original Activity Measures (No time series statistic or DTW measures);
- Model with time series statistic measures + DTW distance measures;
- Model with time series statistic measures only.

Model Configuration: Various hyperparameters configured for the LSTM model are presented in Table 4.

The LSTM models were built using Keras Library with TensorFlow as a backend. All the experiments in this study were conducted in Google Colab, a cloud-based platform for machine learning/deep-learning projects. For this work, run time with a high RAM configuration offered by Google Colab was used. Typical hardware configuration specifications included an Intel Xeon Processor with four cores @ 2.2 GHz and 13 GB RAM. The installed version of Python was 3.10.12, and Keras was 2.13.1.

Model evaluation and metrics: To evaluate the prediction performance of the proposed LSTM model on the test dataset, this study adopted a commonly used metric, “overall prediction accuracy”, as well as three additional, custom-defined metrics, namely, “degenerating identification accuracy”, “transition identification accuracy”, and “MCI identification accuracy”. Accuracy is the measure of various models’ performance. It indicates the extent by which the “labels” (or cognitive stages) are predicted correctly (in other words, Predicted Labels vs. Actual Labels). Testing/prediction accuracy indicates the model’s performance on unseen data (test dataset set aside from the overall dataset). Overall accuracy indicates how many predicted cognitive stage labels match the actual cognitive stage labels among the total test set (i.e., healthy and degenerating cases). Degeneration identification accuracy (DIA) indicates how many predicted cognitive stage labels match the actual cognitive stage labels among the degenerating cases (i.e., TR and MCI). Transition identification accuracy (TIA) indicates how many of the predicted cognitive stage labels match the actual cognitive stage labels among the “TR” cases (TR is an indication of degeneration). MCI identification accuracy (MIA) indicates how many of the predicted cognitive stage labels match the actual cognitive stage labels among the “MCI” cases (MCI is the early stage of degeneration). To compare the LSTM model’s performance against the baseline or traditional classifiers, this study trained and tested Support Vector Machines (SVM) and Random Forest (RF) models.

LSTM Models are examples of “Black Box” models in that the predicted outcomes are not easily understandable in terms of the model inputs or parameters. They require special methods to explain why the model has reached certain conclusions. These special methods help to determine those features that the model considers to be important and explain how these features contribute to the model decision. “Shapley Additive exPlanations”, or simply SHAP, is an algorithm that enables the analysis of the output of models like RNN LSTM [32]. It includes methods to calculate Shapley values—a concept in cooperative game theory. As in cooperative game theory, the SHAP algorithm calculates the “Shapley” value for each feature (or player) and helps to find out how each feature (player) contributed to obtaining that prediction (score) [33]. These values are used for ranking the features and visualizing the important relationships. SHAP offers both “Global Interpretation” (explanation for the entire model behavior) and “Local Interpretation” (explanation for the individual sample/instance prediction). This study adopted the SHAP framework to explain the proposed LSTM model’s predictions and utilized the Python-based SHAP package for this purpose [34].

## 3. Results

In this study, three major modeling scenarios were considered, and several experiments were conducted in each of these scenarios. These three scenarios were differentiated by the set of input features used to train the LSTM model. In the first scenario, model features included the original activity features and demographics (as in Table 2); in the second scenario, model features included the TS statistics, which were extracted from original activity features (as in Table 3), and demographics; in the third scenario, TS statistic features, DTW distance measures, and demographics were all used in the model. In reference to TS statistics, it should be noted that each activity feature was transformed into six TS statistic features (as in Equation (5)) and an example would be as follows: sleep_time_total (original activity measure) > sleep_time_total _mean, sleep_time_total _median, sleep_time_total _stddv, sleep_time_total _varcoeff, sleep_time_total _skew, sleep_time_total _kurtosis.

To understand the significance of TS statistics as features in the model’s prediction performance against the original activity measures, these scenarios were conducted, and the results were compared. In addition, to analyze and understand the impact of various activity categories on the LSTM model’s performance, several experiments were conducted with various combinations of activity categories. In each experiment, models were run five times, and results were averaged to account for the stochastic nature of the algorithms. This study utilized the TensorFlow Keras framework for the LSTM modeling tasks. Results from various experiments in scenario 1—original activity features as inputs—are presented in Table 5. Results from various experiments in scenario 2—TS statistics features and demographics as inputs—are presented in Table 6. Results from various experiments in scenario 3—TS statistics features, DTW distance features, and demographics as inputs—are presented in Table 7. Models with various combinations of activity feature categories were experimented with. Results are averaged from five runs of each mode and stated in descending order of overall accuracy, DIA, TIA, and MIA.

From Table 5, Table 6 and Table 7, it can be noted that the LSTM model trained with TS statistic features, DTW distance features, and demographics (scenario 3) is the best-performing model scenario among all three scenarios. The overall prediction accuracy of 83.84% from scenario 3 is higher than the overall accuracy of 63.37% from scenario 1 and the overall accuracy of 75.53% from scenario 2. A key goal of this study is to identify the degeneration as early as possible, utilizing the activity trends, and in the context of this goal, the accuracy of predicting the degenerative stages (TR or MCI) is quite important. Hence, the accuracy measures DIA, TIA, and MIA play a critical role in analyzing the model’s prediction skill. The results in Table 5, Table 6 and Table 7 show that the degenerating identification accuracy (DIA) from scenario 3 is 80.55% higher than values of 62.7% and 78.2% obtained from scenarios 1 and 2, respectively. Since the cognitive label “TR” indicates the “transitioning”, in other words, the beginning of the transition to MCI, the accuracy measure TIA is also equally important. The model that provides a higher value in both overall prediction as well as degeneration prediction indicates its ability to classify both the healthy and degenerating subjects correctly to a larger extent, and hence, it is helpful to compare TIA across such models that performed well in both overall and degenerating classification. From this perspective, results from scenario 3 show a higher TIA of 78.33% compared to 52.81% and 68.71% from scenarios 1 and 2, respectively (first row in Table 5, Table 6 and Table 7). Summing up the above findings, it is evident that scenario 3 exhibits the best performance in predicting the cognitive stage/label at a future time point and confirms that the transformation of original activity features into a new feature space comprising the “TS statistic” measures as well as “DTW distance” measures provide significant context and longitudinal temporal information to the LSTM model.

To confirm if there exists a statistically significant difference between the prediction performance of scenario 3 and other scenarios (1 and 2), this study adopted the Wilcoxon Signed-Rank Test with statistical significance, *p* < 0.05. The top five models from scenario 3 (Table 7) were selected, and a statistical significance test was conducted for all accuracy metrics. Table 8 provides another view of accuracy (performance) metrics across all the models, along with the statistical significance. From Table 8, the majority of scenario 3’s performance demonstrates a statistically significant difference from other scenarios. To understand whether the LSTM model performs better than traditional classifiers, classifiers, namely, SVM and RF models, were trained with TS statistics, DTW distance, and demographics features (as in scenario 3). Note that these features belonged to all activity categories. In addition, the Wilcoxon Signed-Rank Test was conducted to confirm the performance of the model, showing a statistically significant difference. From the results listed in Table 9, the LSTM model performed far better than these traditional/baseline classifiers.

## 4. Discussion

To the best of our knowledge, this study is the first of its kind to examine the use of continuously acquired daily activity data in deriving appropriate digital biomarkers, recognizing temporal patterns, and predicting the cognitive stage shift at a future time point. Many of the studies in this field of research focus on leveraging multi-modal data usually obtained from invasive methods. However, the results from this study suggest an inexpensive, non-invasive approach to gathering activity data could potentially be an alternate approach in the field of AD progression modeling, specifically, transition to MCI. Results from various experiments demonstrate that the prediction performance (overall and specifically degeneration identification) is superior when activity trends are represented as time series statistics and distance measures compared to activity measures represented in original numerical values form. This means that the LSTM model can effectively learn the activity pattern changes occurring over time, including the temporal connections across various time points. This is, in fact, addressing one of the research questions framed in this study.

Another observation from these results is the LSTM model performs better when all activity feature categories (walk, sleep, dwell, out-of-home) are combinedly given as input, compared to when the activity feature categories are given individually. The combination of all activity feature categories gives a holistic picture of the older adults’ daily routines, and that helps the LSTM model learn the finer details of their activity trends, particularly the association with cognitive decline, as seen from the higher DIA reported.

From all three scenario results of LSTM experiments, it can be observed (Table 5, Table 6 and Table 7) that, in top-performing models, the “sleep” activity category is inevitably present, either individually or combined with another activity category (e.g., “walk, sleep, and OTH”, “sleep and dwell’, “sleep and walk”). This observation indicates that the “sleep”-related measures are the key indicators for identifying the degeneration or MCI transition. A deep look at the results from the top five models in scenario 3 (Table 7) reveals that only a marginal difference exists between the “sleep only” model and the rest of the top four model results. This essentially indicates that the features from the “sleep” activity category alone can provide sufficient information to the LSTM model to understand the underlying degeneration process. These findings on sleep activity’s impact in this study are corroborated by findings from other studies in the literature, such as Xu et al. [35]. Their study concluded that various sleep-related parameters were linked to an increased risk of cognitive decline. Chen et al. [36] found a statistically significant association between sleep duration and a decline in cognitive functions.

**Figure 6:**
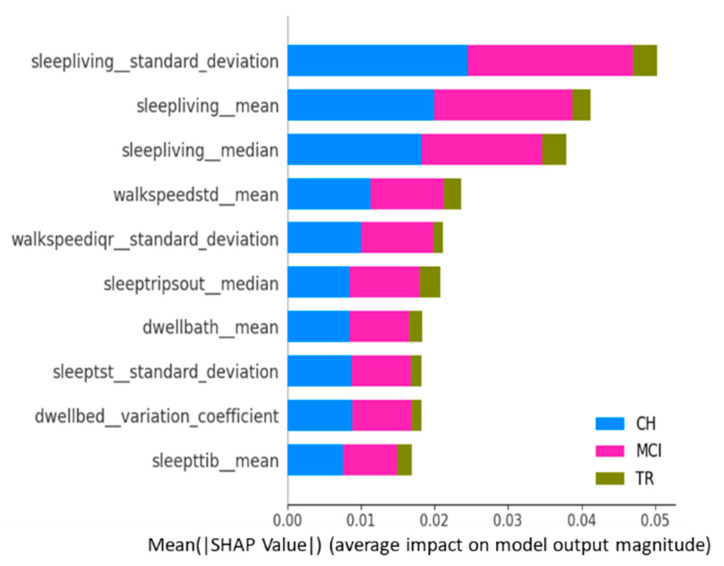
Global interpretation with SHAP values: top 10 features—all target classes combined.

All the above explanations or findings are at the activity category level. It is important to explain the results further at the individual feature level. As stated in the methods section, this study adopted the SHAP framework to identify which individual features contributed most to the predictions and help explain whether these explanations are clinically relevant. Feature importance is determined in terms of SHAP values produced by this framework. For this SHAP explanation, this study leveraged the best performing LSTM model (i.e., all activity categories model from scenario 3). Figure 6 is the summary plot consisting of SHAP values from all target classes/labels. This summary plot lists the top 10 features contributing to the model prediction overall (either positively or negatively to the target variable) [32] and, thus, provides a “global interpretation” of the entire model behavior. This plot shows the average impact each activity feature has on predicting each cognitive stage, regardless of whether it is positive or negative.

**Figure 7:**
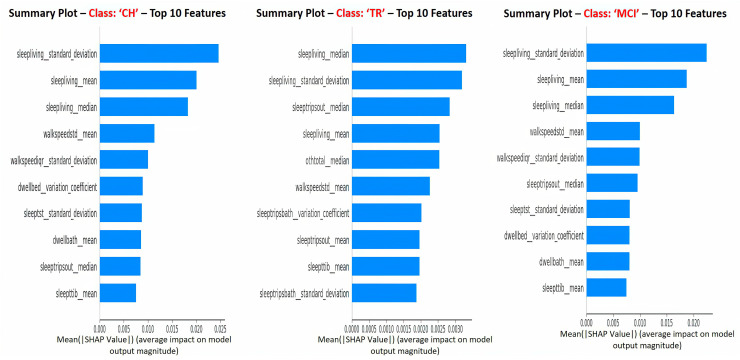
Global interpretation with SHAP values: top 10 features—plots for individual target classes.

Figure 7 depicts the SHAP summary plots for each of the target classes separately, and these plots correspond to the global interpretation of the model behavior. From these global interpretation plots, the following observations can be made:

- Sleep activity-related features (e.g., sleep duration in the living room, total sleep time in bed, number of trips out during sleep time, and total sleep duration) occupy 60–70% of the list of top 10 important features across various target classes. This is in alignment with explanations given at the “sleep activity category” level in previous paragraphs and confirms the significance of sleep features as key predictors;
- Sleep-related features in this context indicate the quality of sleep, including sleep disturbances, variation in sleep durations, as well as quantity of sleep;
- In addition, TS statistics such as “mean”, “median’, and “standard deviation” values came out to be present in the top 10 features that drive the model predictions (predict the cognitive stage at future time point). These TS statistic features measured over time on sleep activity indicate the changes in sleep patterns, which could relate to both sleep quality and quantity. These observations from this study are in alignment with other studies that found the impact of sleep patterns on cognitive decline/degeneration. For example, Ju et al. [37] found that sleep quality/efficiency was associated with the presence of biological markers of AD to indicate the preclinical stage of AD. Diem et al. [38] observed that variability in sleep efficiency (quality) and total sleep time (quantity) were associated with the increased risk of developing cognitive impairment;
- “Sleep Living Mean” and “Sleep Living Median” represent the center point or operating point of this activity data, around which most of the data points cluster. The trend in these center values over time (in other words, the changes in the quantum of sleep duration in the living room over time) greatly influences the prediction of the next cognitive stage. On the other hand, the feature “Sleep Living Standard Deviation” represents the variability or fluctuations of the “sleep duration in the living room” over time. This variability is also one of the influencers in predicting the cognitive stage, and it possibly indicates the changes in the patterns of sleep and, hence, the anomalies;
- The next set of activity features in the top list corresponds to “walking speed”. The mean value (or operating level) and fluctuations in walking speed play an important role in the model prediction. This finding is in alignment with other studies’ outcomes in the literature, such as Kaye et al. [39] and Dodge et al. [40], who found walking speed and related measures to be strong indicators in predicting cognitive/functional health in older adults;
- In addition to sleep and walk features, features such as dwell time in the bathroom and bedroom areas are also found to be in the top 10 list. Higher dwell time in a specific space could indicate a lack of movement; on the other side, lower dwell time could indicate the miss of the action/function associated with that space. For example, higher dwell time in the bedroom (day + nighttime) could mean no movement during the day or active functioning; lower dwell time in the bathroom could mean that the subject did not take a shower or make use of the toilet; either scenario could be a result of forgetfulness, which is the key issue in MCI.

The results (accuracy metrics and interpretations) from this study appear promising in the early detection of the cognitive stage transition. In real-life clinical settings, the proposed method can be used as an initial screening method for early detection of the cognitive stage transition with further follow-ups and personalized intervention plans by clinicians. Since this study corresponds to an emerging field of research, acceptance by the medical community as a diagnostic tool over the traditional methods in clinical practice will evolve over time, and that will depend on the reliability and generalizability of the proposed method compared to the traditional diagnostic/assessment methods. The results demonstrate that this study is certainly a stepping stone for further investigations.

Soon, other deep-learning architectures, such as autoencoders and transformers, will be leveraged in modeling the AD progression. Prediction of cognitive stages at multiple future time points (prediction window) will be investigated, and model performance will be compared for various observation and prediction window combinations.

## 5. Conclusions

The LSTM model trained with TS statistic measures and DTW distance measures outperformed various scenarios with an overall prediction accuracy of 83.84%. Furthermore, this model scenario exhibited better performance than traditional classifiers in terms of metrics, such as degeneration identification accuracy (80.55%), transition identification accuracy (78.33%), and MCI identification accuracy (82.60%). In summing up all the above observations, this study finds that the proposed LSTM model leveraging older adults’ daily routines captured through continuous monitoring can predict the MCI transition at a future time point. Also, this study can demonstrate the activity features when they are transformed from original numerical values to time series statistic measures, provide valuable information regarding temporal dependencies in the sequential activity data, and, thus, improve the prediction performance. Intra-individual activity patterns and inter-individual activity patterns are represented effectively using TS statistic measures and DTW distance measures, respectively. This study finds these representations very effective in improving the LSTM model’s prediction skill in comparison to traditional classifiers. Based on the results of this study, “sleep”-related activity features are found to be the key indicators for cognitive decline or degeneration, and many studies in the literature have found the sleep measures to be clinically relevant in predicting cognitive dysfunction or decline. The results from this study suggest that continuously acquired daily activity data via inexpensive, non-invasive, and ubiquitous ambient sensors can provide sufficient digital biomarkers to predict the MCI transition at a future point.

## Figures and Tables

**Table 1:** Characteristics of subjects in the study dataset.

Transition Scenario	Count	Age at the Baseline, Years, Mean (SD)	Education, Years in School, Mean (SD)	Mean Sensor Monitoring Duration, Months	MMSE Score at Baseline, Mean (SD)
CH	Male, *n* = 10	81.91 (6.12)	17.2 (1.75)	33.86	28.9 (1.37)
	Female, *n* = 37	81.85 (7.67)	15.35 (2.31)	33.13	29.27 (0.87)
CH- > TR	Female, *n* = 52	86.77 (9.82)	14.25 (2.51)	28.62	28.5 (1.13)
CH- > TR- > MCI	Female, *n* = 50	80.25 (12.58)	14 (0)	30.60	29.5 (0.51)
MCI	Male, *n* = 24	85.58 (4.8)	17.75 (2.33)	25.24	27.75 (0.85)
	Female, *n* = 24	92.03 (2.12)	13.75 (1.51)	28.11	27.5 (1.69)
TR	Female, *n* = 50	85.15 (0.66)	17 (1.01)	11.61	28 (1.01)
TR- > MCI	Male, *n* = 25	97.7 (0)	13 (0)	18.45	27 (0)
	Female, *n* = 25	86.7 (0)	16 (0)	30.52	30 (0)

**Table 2:** Original activity measures and demographic features [10].

Cognitive/Behavioral Domain	Activity Category	Activity Measure/Feature
Demographics	Demographics	Gender Years of education Ethnicity
Physical Mobility	Dwell	Dwell time in bathroom area in seconds Dwell time in bedroom area in seconds Dwell time in living room area in seconds
Social engagement	OTH	Out-of-home (OTH) number of instances Out-of-home (OTH) total time seconds
Sleep and nighttime behavior	Sleep	Sleep in living room in seconds Number of trips out of bedroom (sleep time) Sleep time total in seconds Wake after sleep onset (WASO) in seconds Sleep latency
Physical Mobility	Walk	Walking speed variability Walking speed (cm/second) Number of captured walks

**Table 3:** Time series statistic features.

TS Statistic Feature	Significance	Indication
Median	Level of data/operating point	Measure of central tendency
Mean	Level of data/operating point	Measure of central tendency
Standard deviation	Fluctuations in the level of data (i.e., stability)	Measure of variability
Variation coefficient	Degree of fluctuation with respect to mean (i.e., consistency)	Measure of variability
Skewness	Asymmetry of the data	Shape of distribution
Kurtosis	Peaked Ness of the distribution	Shape of distribution

**Table 4:** LSTM model configuration.

Parameter	RNN-LSTM
Hidden layers	3
Neurons (units) in first layer	100
Neurons (units) in second layer	64
Neurons (units) in third layer	16
Activation	SoftMax
Optimizer	Adam
Learning rate	0.0001
No of epochs	150
Dropout rate	0.5

**Table 5:** Results of scenario 1—LSTM model trained with original activity features and demographics. Models with various combinations of activity feature categories were experimented with. Results are averaged from 5 runs of each model and stated in descending order of overall accuracy, DIA, TIA, and MIA.

Model	Overall Accuracy	DIA	TIA	MIA
All Activity Categories	63.37%	62.70%	52.81%	71.87%
Walk, Sleep, and OTH	59.60%	58.65%	43.86%	72.36%
Walk and Sleep	55.89%	57.22%	44.56%	68.94%
Sleep and OTH	51.18%	46.27%	46.78%	45.80%
Sleep and Dwell	49.94%	43.88%	48.54%	39.57%
Sleep Only	42.36%	35.86%	44.91%	27.48%
Dwell Only	38.65%	47.59%	98.95%	0.00%
Walk and Dwell	37.91%	36.29%	60.00%	14.31%
Walk and OTH	37.71%	33.50%	64.56%	4.72%
OTH and Dwell	36.70%	36.43%	75.73%	0.00%
OTH only	36.70%	42.36%	88.07%	0.00%
Walk Only	34.55%	28.52%	50.70%	7.97%

**Table 6:** Results of scenario 2—LSTM model trained with TS statistics features and demographics. Models with various combinations of activity feature categories were experimented with. Results are averaged from 5 runs of each mode and stated in descending order of overall accuracy, DIA, TIA, and MIA.

Model	Overall Accuracy	DIA	TIA	MIA
Walk, Sleep, and OTH	75.53%	78.20%	68.71%	86.99%
All Activity Categories	75.49%	75.78%	71.23%	80.00%
Walk and Sleep	71.78%	73.59%	59.82%	86.34%
Sleep and OTH	67.68%	69.62%	71.64%	67.75%
Sleep and Dwell	63.86%	60.62%	62.57%	58.81%
Sleep Only	60.47%	60.76%	58.60%	62.76%
Walk and OTH	58.86%	60.68%	70.00%	52.03%
Walk and Dwell	55.56%	56.54%	59.65%	53.66%
OTH only	38.72%	44.56%	92.63%	0.00%
OTH and Dwell	34.12%	31.50%	39.18%	24.39%
Walk Only	30.98%	21.52%	34.21%	9.76%
Dwell Only	30.77%	30.46%	60.00%	3.09%

**Table 7:** Results of scenario 3—LSTM model trained with TS statistics features and demographics. Models with various combinations of activity feature categories were experimented with. Results are averaged from 5 runs of each mode and stated in descending order of overall accuracy, DIA, TIA, and MIA.

Model	Overall Accuracy	DIA	TIA	MIA
All Activity Categories	83.84%	80.55%	78.33%	82.60%
Sleep and Dwell	81.87%	78.41%	78.80%	78.05%
Sleep and OTH	81.62%	78.40%	81.93%	75.12%
Walk, Sleep, and OTH	81.48%	77.72%	78.60%	76.91%
Sleep Only	80.44%	76.75%	77.89%	75.69%
Walk and Sleep	79.76%	75.49%	73.68%	77.15%
Walk and Dwell	75.29%	71.52%	73.86%	69.35%
Walk and OTH	75.29%	70.59%	77.89%	63.82%
Walk Only	68.62%	62.87%	73.95%	52.60%
OTH Only	29.29%	23.95%	49.78%	0.00%
Dwell Only	23.64%	10.21%	21.23%	0.00%
OTH and Dwell	21.55%	3.88%	0.00%	7.48%

**Table 8:** Consolidated view of all accuracy metrics obtained from top 5 models across various scenarios. Models in the top 5 positions from scenario 3 (S3) are taken as the reference for this comparison.

Model	Overall Accuracy			DIA			TIA			MIA		
	S1	S2	S3	S1	S2	S3	S1	S2	S3	S1	S2	S3
ALL Features	63.37%	75.49%	83.84% *^#^	62.70%	75.78%	80.55% *^#^	52.81%	71.23%	78.33% *^#^	71.87%	80.00%	82.60% *
Sleep and Dwell	49.94%	63.86%	81.87% *^#^	43.88%	60.62%	78.41% *^#^	48.54%	62.57%	78.80% *^#^	39.57%	58.81%	78.05% *^#^
Sleep and OTH	51.18%	67.68%	81.62% *^#^	46.27%	69.62%	78.40% *^#^	46.78%	71.64%	81.93% *^#^	45.80%	67.75%	75.12% *^#^
Walk, Sleep, and OTH	59.60%	75.53%	81.48% *^#^	58.65%	78.20%	77.72% *	43.86%	68.71%	78.60% *^#^	72.36%	86.99%	76.91% ^#^
Sleep Only	42.36%	60.47%	80.44% *^#^	35.86%	60.76%	76.75% *^#^	44.91%	58.60%	77.89% *^#^	27.48%	62.76%	75.69% *^#^

* Statistically significant performance improvement compared to scenario 1 (S1) models (Wilcoxon test, *p* < 0.05). ^#^ Statistically significant performance improvement compared to scenario 2 (S2) models (Wilcoxon test, *p* < 0.05).

**Table 9:** Performance comparison between LSTM and traditional classifiers.

Activity Categories Included	Scenario	Model	Overall Accuracy	DIA	TIA	MIA
All Activity Categories	Scenario 3 (TS statistic, DTW distance, and demographic features)	LSTM	83.84% *^#^	80.55% *^#^	78.33% *^#^	82.60% *^#^
		SVM	60.00%	55.56%	60.00%	50.00%
		RF	60.00%	55.56%	40.00%	75.00%

* Statistically significant performance improvement compared to SVM model (Wilcoxon test, *p* < 0.05). ^#^ Statistically significant performance improvement compared RF model (Wilcoxon test, *p* < 0.05).

## Data Availability

The authors would like to acknowledge the Alzheimer’s Disease Center Clinical Core (PI: J. Kaye; eIRB 725; supported by the National Institute on Aging P30 AG008017) and the Oregon Center for Aging and Technology (ORCATECH; PI: J.Kaye; eIRB 2765; supported by the National Institute on Aging P30 AG024978) for supporting with the dataset for this study. Researchers needing access to this dataset may contact ORCATECH by filling out the data request form (https://www.ohsu.edu/oregon-center-for-aging-and-technology/data-resources, accessed on 3 September 2023).
